# Papain Covalently Immobilized on Chitosan–Clay Nanocomposite Films: Application in Synthetic and Real White Wine

**DOI:** 10.3390/nano10091622

**Published:** 2020-08-19

**Authors:** Ilaria Benucci, Claudio Lombardelli, Ilaria Cacciotti, Marco Esti

**Affiliations:** 1 Department of Agriculture and Forestry Science (DAFNE), Tuscia University, via S. Camillo de Lellis snc, 01100 Viterbo, Italy; ilaria.be@unitus.it (I.B.); claudiolombardelli@libero.it (C.L.); esti@unitus.it (M.E.); 2 Department of Engineering, University of Rome “Niccolò Cusano”, INSTM RU, Via Don Carlo Gnocchi, 3, 00166 Rome, Italy

**Keywords:** chitosan, clay, nanocomposite films, papain, covalent immobilization, wine

## Abstract

Increasing attention has been recently paid to the development of nanocomposite materials for food application as new tool to enhance the mechanical and thermal properties of polymers. In this study, novel chitosan–clay nanocomposite films were produced as carriers for the covalent immobilization of papain, by using a fixed amount of chitosan (1% *w*/*v*) and a food-grade activated montmorillonite (Optigel, OPT) or a high-purity unmodified montmorillonite (SMP), in four different weight percentages with respect to chitosan (i.e., 20, 30, 50, 70% *w*/*w*). Both nanoclays (OPT and SMP) improved the mechanical properties of the obtained nanocomposites, and the OPT films showed the highest Young modulus and mechanical resistance (σ_max_). The nanocomposites were used as carriers for the covalent immobilization of papain, which was preliminarily characterized in model wine towards a synthetic substrate, showing the highest efficiency in the release of the reaction product when it was bound on OPT-30 and OPT-50 films. Finally, the latter biocatalyst (papain on OPT-50 film) was applied for the protein stabilization of two different unfined white wines, and it efficiently reduced both the haze potential and the protein content.

## 1. Introduction

The application of enzymes as powerful catalysts in a large number of industrial fields, including pharmaceuticals and food, allows the development of efficient processes with low environmental impact [1]. In the wine-making industry, some enzymes have been traditionally used whereas others have been only recently introduced. Proteases, able to catalyze the hydrolysis of proteins, have been proposed as a selective alternative to the conventional bentonite fining for removing the haze-forming proteins, which are responsible for haze formation in white wine under post-bottling conditions.

In recent decades, immobilization techniques have been successfully applied to enhance enzyme properties, in particular stability and reusability [2]. The material employed for the support preparation is of fundamental importance in the immobilization process, affecting the catalytic properties of the produced biocatalyst. A wide range of inorganic and organic materials, as well as composites, may be applied as carriers for enzyme immobilization [3,4] in different shapes (e.g., films [5], beads [6,7] and fibers [8]). Recently, increasing attention has been paid to organic–inorganic composite materials for food application. Regarding composite films as supports for enzyme immobilization, Yang et al. [9] reported that the chemical and thermal stability of organic membranes could be enhanced by an inorganic phase. The latter usually allows the stabilization of enzyme–support interactions [10].

Among biopolymers, chitosan (CS, a linear polysaccharide made of *N*-acetyl-d-glucosamine and d-glucosamine units [11]) is the most commonly used for the production of immobilization supports as it is, as well as in composite/nanocomposite forms [2], to be applied in the food and pharmaceutical industries.

Several studies have been aimed at improving the mechanical and thermal stability of neat CS films [12,13,14] by seeking CS-based nanocomposites, with the addition of specific nanoparticles to enhance the aforementioned properties [3,13,15]. The nanomaterials dispersed within the chitosan matrix, e.g., nanoclays, carbon structures, and metal/metal oxides, via physical or chemical interaction [16], allow not only to enhance the physical, mechanical, and thermal stability but also to endow the composite with their intrinsic features, e.g., high specific surface area [11]. 

Among the nanoparticles, clay is a fine-grained soil material that contains metal oxides or hydroxides with traces of organic matters. Due to its smallest dimension, excellent colloidal properties [11], and ability to interact with CS by electrostatic force, clay has been used for the preparation of CS–clay nanocomposites through adsorption, gelation, or intercalation [13,17]. 

Recently, CS–clay nanocomposite films have been prepared by using different nanoclays (i.e., bentonite, sepiolite and montmorillonite (MMT)) and have been applied as supports for the covalent immobilization of stem bromelain in wine production [13,15]. In our previous works, we proposed nanocomposites based on CS and nanoclays in very low concentrations (1–5% *w*/*w*) [12] and systems based on CS and nanoclays in high concentrations (70–80% *w*/*w*) [14], maintaining, in both cases, the total weight of the composite constant. Papain from Carica papaya L. latex (EC 3.4.22.2), a cysteine protease applied in brewing for the removal of chill haze [18] and for the clarification treatment of pomegranate juice [19], has been covalently immobilized on CS. Moreover, Benucci et al. [20] immobilized papain on commercial chitosan beads by direct linkage for the protein stabilization of white wines. 

This study was focused on the production of novel CS–clay nanocomposite films, using a fixed amount of CS (1% *w*/*v*) and a food-grade activated MMT (Optigel, OPT) or a high-purity unmodified MMT (SMP), in four different weight percentages with respect to CS (i.e., 20, 30, 50, 70% *w*/*w*), as carriers for the covalent immobilization of papain. 

The physical properties, in terms of the morphology, thermal stability and the mechanical behavior, of the produced nanocomposites were investigated. Finally, the catalytic properties of immobilized papain were tested in synthetic wine and its effectiveness in protein stabilization was investigated in real white wines.

## 2. Materials and Methods 

### 2.1. Enzyme, Chemicals and Wines

Papain from Carica Papaya latex (EC 3.4.22.2), shellfish-derived chitosan (CS) powder (low molecular weight 50–190 kDa; percentage of deacetylation 75%), glutaraldehyde (GDH, 25% v/v), and glycerol (≥99.5%) were obtained from Sigma-Aldrich (Milan, Italy). Two different nanoclays, i.e., activated food-grade montmorillonite (Optigel, OPT) and high-purity unmodified montmorillonite (SMP), were kindly provided by BYK Additives GmbH (Wesel, Germany) and Zhejiang Fenghong New Material Co., Ltd. (Huzhou, China), respectively. The selected synthetic tripeptide chromogenic substrate (Bz–Phe–Val–Arg–p-nitroaniline (pNA)), applied for the kinetic characterization of immobilized papain, was purchased from Bachem (Bubendorf, Switzerland). All the other chemicals were of analytical grade (Sigma Aldrich, Milan, Italy). Manzoni and Sauvignon Blanc unfined white wines (vintage 2018) were kindly provided by Casale del Giglio winery (Le Ferriere, LT, Italy) and their oenological parameters are summarized in Table 1. 

### 2.2. Preparation of CS–Clay Nanocomposite Films by Solvent Casting

CS–clay supports were obtained by solvent casting technique, using low molecular weight CS (1% *w*/*v*) blended with glycerol (CS:glycerol 75:25, in %*w*/*w*) [5], and adding two different nanoclay types, i.e., OPT and SMP, in four different weight concentrations (i.e., 20, 30, 50, 70% *w*/*w*). The set-up procedure was reported elsewhere [5]. Briefly, nanoclays aqueous suspensions were prepared by ultrasonication using Sonics Vibracell CV33 (Sonics & Materials Inc., Newtown, CT, USA) in the following conditions: power 750 W, frequency 20 kHz, amplitude 30%, time 30 min. Afterwards, acetic acid (2% *v*/*v*), glycerol and CS were added and the mixtures were maintained under continuous magnetic stirring overnight. The final suspensions were solvent cast onto plastic Petri dishes, in a fume hood, and dried at room temperature for 48 h. Furthermore, as a reference, a system composed of only CS and glycerol was produced. The obtained samples were designed as Clay-free for the CS/glycerol-based film and OPT-*x* and SMP-*x* in the case of clay-loaded composites (where *x* is the w/w percentage of the used nanoclay with respect to the CS, i.e., 20, 30, 50, and 70% *w*/*w*).

### 2.3. Physical Characterization of CS–Clay Nanocomposite Films

The produced films were morphologically, thermally and mechanically analyzed by Field-Emission Gun Scanning Electron Microscope (FEG-SEM, Leo Supra 35, Carl Zeiss SMT Ltd., Cambridge, UK), differential scanning calorimetry (DSC, TAInstruments Q2000, New Castle, DE, USA) and uniaxial tensile tests (Lloyd LRX, Lloyd Instruments Ltd., West Sussex, UK), respectively. In detail, the SEM micrographs were acquired on gold-coated samples, applying a voltage of 3–5 kV. DSC measurements were carried out between 25 and 400 °C (heating rate 10 °C·min^−1^), in a nitrogen atmosphere (N_2_ flow rate 50 cc·min^−1^), using a sample weight of ~5 mg. For the mechanical investigation, the ASTM D1708 and ASTM D882 standards were followed, and the nominal specimen cross-section was considered for the measurement of all mechanical features. Dog-bone specimens (width 4.8 mm, length 22.25 mm) were obtained from the produced films. The tests were performed at 1.2 mm·min^−1^, using a 50 N load cell. All measurements were taken in triplicate.

### 2.4. Papain Immobilization on CS–Clay Nanocomposite Films

Before enzyme immobilization, the CS–film samples were neutralized by overnight shaking in 26% (*v*/*v*) ethanol–NaOH 2 M solution and then cut into squares (10 mm × 10 mm) with a razor blade. The immobilization procedure was performed as briefly reported in the following [13]. The film surface was activated by adding 1 mL of 3% (*v*/*v*) GDH as a cross-linker, and keeping at room temperature under constant agitation (120 rpm) for 2 h. The activated films were thoroughly washed with distilled water; thereafter, 1 mL of papain preparation (0.12 mg_protein_/mL solubilized in the tartaric buffer) was added. After the overnight incubation (150 rpm at 20 °C), the obtained biocatalysts were carefully washed with tartaric buffer, and then left to stand for 20 min in 0.1 M glycine solution. At the end of the immobilization procedure, the biocatalysts were washed three times with 2 M NaCl solution to remove all non-covalently bound proteins. The immobilization yield (IY, %) was calculated as the difference between the protein concentration in the enzyme solution, before and after immobilization. The protein concentration was evaluated by the Bradford method [21] using bovine serum albumin (BSA) as a standard protein.

### 2.5. Proteolytic Activity Assay

Proteolytic activity toward the tripeptide chromogenic substrate (Bz–Phe–Val–Arg–pNA) was tested in model wine (0.03 M, tartaric acid/sodium tartrate solution pH 3.2, containing 12% *v*/*v* of ethanol) at 20 °C, as reported by Benucci et al. [13].

### 2.6. Kinetic Characterization of Immobilized Papain

A kinetic study of papain immobilized on CS–clay nanocomposite films was carried out in model wine, fortified with Bz–Phe–Val–Arg–pNA substrate (0–750 μM). Kinetic parameters (V_max_ and K_M_ (Michaelis–Menten constant)) were determined according to the Michaelis–Menten equation using a non-linear regression procedure (GraphPad Prism 5.01, GraphPad Software, Inc., San Diego, CA, USA). Moreover, k_cat_ (turnover number) and K_a_ (affinity constant) were calculated as described by Benucci et al. [13].

### 2.7. Wine Stabilization Treatment in the Batch-Scale Stirred Reactor

Ten milliliters of each white wine were treated using the immobilized biocatalyst in a laboratory-scale stirred reactor (120 rpm, 20 °C) for 24 h. Trials were performed in triplicate, in three different stirred reactors, in order to obtain three independent replicates. To investigate the potential absorption effect of the CS–clay nanocomposite films, a blank correction was made using the supports covered with protease firstly immobilized and then deactivated with NaOH 16 N.

### 2.8. Wine Protein Content Determination

The total protein content of both white wines, before and after the enzymatic treatment in the laboratory-scale stirred reactor, was determined using the potassium dodecyl sulphate method, according to Gaspar et al. [22]. All measurements were made in triplicate.

### 2.9. Heat Test

The protein stability of both white wines, before and after the enzymatic treatment in laboratory-scale stirred reactor, was investigated by heat test, incubating the wines at 80 °C for 6 h, and then keeping them at 4 °C for 16 h [23]. Haze formation was measured using HD 25.2 turbidimeter (Delta Ohm, Padua, Italy), and the turbidity was expressed in nephelometric turbidity units (NTU). Wine stability was calculated as the difference in the wine NTU after and before the heat test (haze potential). The turbidity removal yield (TRY (%)) was expressed as the percentage of haze potential decrease due to the proteolytic treatment. All measurements were made in triplicate.

### 2.10. Statistical Analysis

One-way completely randomized analysis of variance (ANOVA), with an EXCEL^®^ Add-in macro, was employed to analyze the data, obtained from the average of three replicate measurements. Then, a Tukey honestly significant difference (Tukey HSD) post-hoc test (*p* = 0.05) for the multiple comparisons of samples was applied.

## 3. Results and Discussion

### 3.1. Physical Properties of CS–Clay Nanocomposite Films

**Figure 1:**
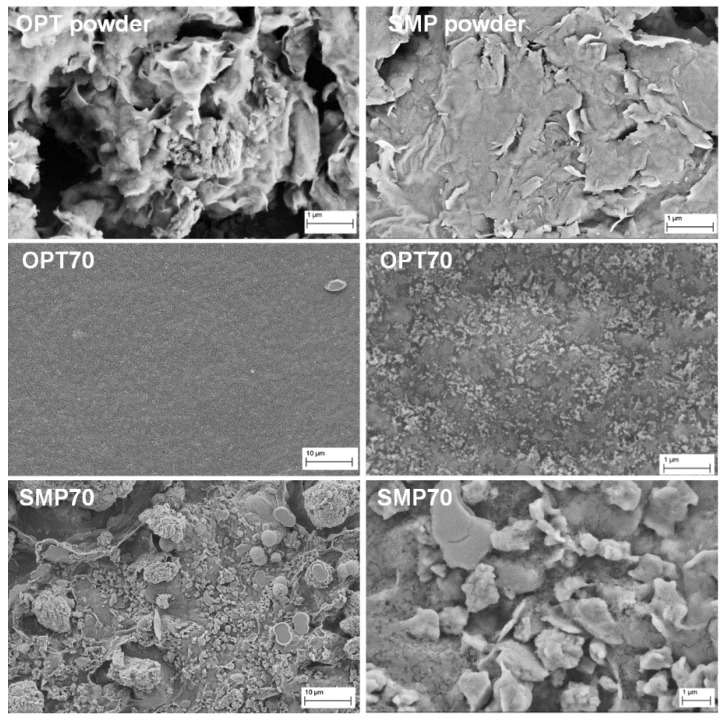
SEM micrographs of the food-grade activated montmorillonite (Optigel, OPT) and high-purity unmodified montmorillonite (SMP) powders, and the OPT- and SMP-based films.

All the samples were morphologically analyzed. As an example, the SEM micrographs of SMP70 and OPT70 are compared in Figure 1. Two very different filler distribution behaviors were observed. OPT clays were better dispersed, promoting a more uniform and homogeneous surface. On the contrary, the SMP addition induced the development of several agglomerates, leading to a very rough surface, characterized by several defects (Figure 1).

**Figure 2:**
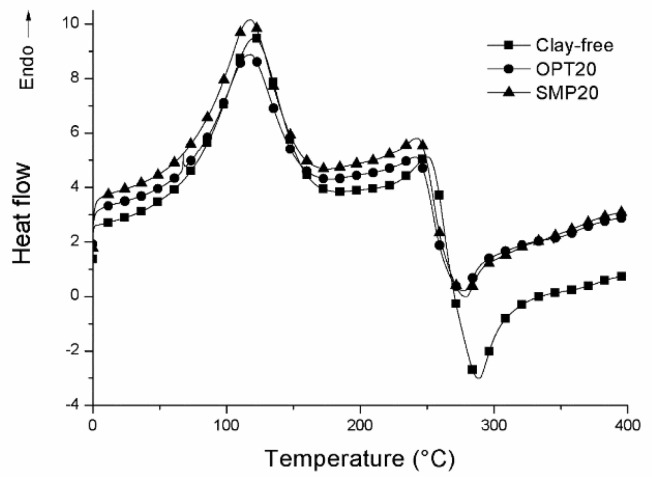
Differential scanning calorimetry (DSC) thermograms of Clay-free, OPT20 and SMP20 samples.

The influence of OPT and SMP on the thermal properties was explored by DSC measurements. In Table 2, the acquired thermal properties are collected and the DSC thermograms of OPT20 and SMP20, as an example, are compared to that of the clay-free sample in Figure 2.

In all cases, two main peaks, one endothermic and the other one exothermic, were detected at approximately 105–120 °C and 235–335 °C (Table 2), respectively. The first one was ascribed to the dissociation process of interchain hydrogen bonding of CS (T_IHB_) [24,25], whereas the second one to CS decomposition (T_d_) [26,27,28]. In all the composite systems, a decrement of T_IHB_ temperature was revealed, compared to Clay-free (121 °C). Particularly, a progressive decrease increasing the nanoclay amount was evidenced in the case of SMP-based films. For the related enthalpies, different trends were recorded for OPT- and SMP-based films. For the former, a decrement of the enthalpy was evidenced increasing OPT amount, whereas, an opposite trend was revealed for SMP-based composites. This experimental evidence indicates that higher energy is necessary in order to induce the dissociation process of interchain hydrogen bonding of CS in the case of SMP-based systems. Concerning the chitosan degradation temperature, a decrease was evidenced in the case of composite systems with respect to Clay-free film, suggesting a not good filler dispersion within the polymeric matrix, with the consequent development of many agglomerates.

**Figure 3:**
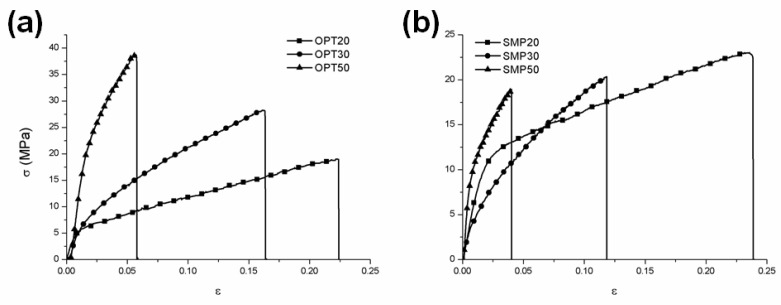
σ–ε curves of OPT (**a**) and SMP (**b**) based films.

The effect of nanoclays on the mechanical behavior was analyzed performing uniaxial tensile tests (Figure 3, Table 3).

An improvement in the mechanical responsiveness, in terms of the Young modulus and maximum stress value (σ_max_), was evidenced in the case of all composite systems, particularly those based on OPT, due to its better distribution within the polymeric matrix, as testified by the reported SEM micrographs (Figure 1) and DSC data (Table 2). The OPT and SMP-based films presented different mechanical behavior. In detail, a progressive increment of the ultimate tensile strength (σ_max_) was evidenced for OPT-based samples with the OPT amount, whereas an opposite trend was observed in the case of SMP-based ones (Table 3), probably due to the presence of several agglomerates whose amount tended to increase with filler percentage (Figure 1), as expected.

Additionally, a remarkable increase in the Young Modulus value was observed for a nanoclay amount of 50% w/w, independently of the considered nanoclay. On the contrary, up to 30% w/w), comparable values were recorded (Table 3). As expected, by increasing the nanoclay amount, a progressive decrease in the elongation at break (ε_max_) was revealed.

Taking into account all the collected morphological, thermal and mechanical properties, it is evident that OPT presented a higher affinity towards CS, as testified by its better distribution within the polymeric matrix, promoting a good filler-polymeric chains interaction.

Indeed, SMP and OPT led to distinct behaviour due to their different chemical nature and microstructure. In details, even if both SMP and OPT powders were composed of platelet-like particles (Figure 1), a higher tendency to agglomeration was evidenced in the case of SMP powder, whereas OPT nanoclays were more homogeneously dispersed within the polymeric matrix. These experimental evidences could be ascribed to OPT good wettability and compatibility, able to promote a significant improvement of the mechanical behavior (Table 3).

### 3.2. Kinetic Properties of Papain Immobilized on CS–Clay Nanocomposite Films

CS–clay films, produced by the addition of two nanoclays (OPT and SMP) in different amounts (20, 30, 50, 70% *w*/*w* with respect to CS), were applied as supports for the papain covalent immobilization.

Data in Table 4 show that the IY was significantly higher for SMP-70 (60%) with respect to Clay-free sample (45%), thus proving that the addition of such nanoclay remarkably increased the amount of covalently bound protein, probably due to its higher surface roughness and better affinity between SMP clay and the papain. Similarly, Basak et al. [29] demonstrated that the addition of bentonite into chitosan beads (ratio 50:50% *w*/*w*) significantly increased the catalase immobilization yield. Furthermore, the addition of OPT nanoclay significantly decreased the IY, with the only exception of OPT-70. These data appeared consistent with those described by Cacciotti et al. [15], who found that the IY slightly decreased when the bromelain from pineapple stem was immobilized on nanocomposites obtained combining MMT (i.e., SMP and OPT) with low amounts of CS in different relative ratios (MMT:CS 70:30, 75:25 and 80:20, % *w*/*w*), compared to Clay-free support. Moreover, the lowest IY was found for OPT-30 and OPT-50 films (26 and 29%, respectively), whereas no relevant differences were revealed comparing the other samples.

**Figure 4:**
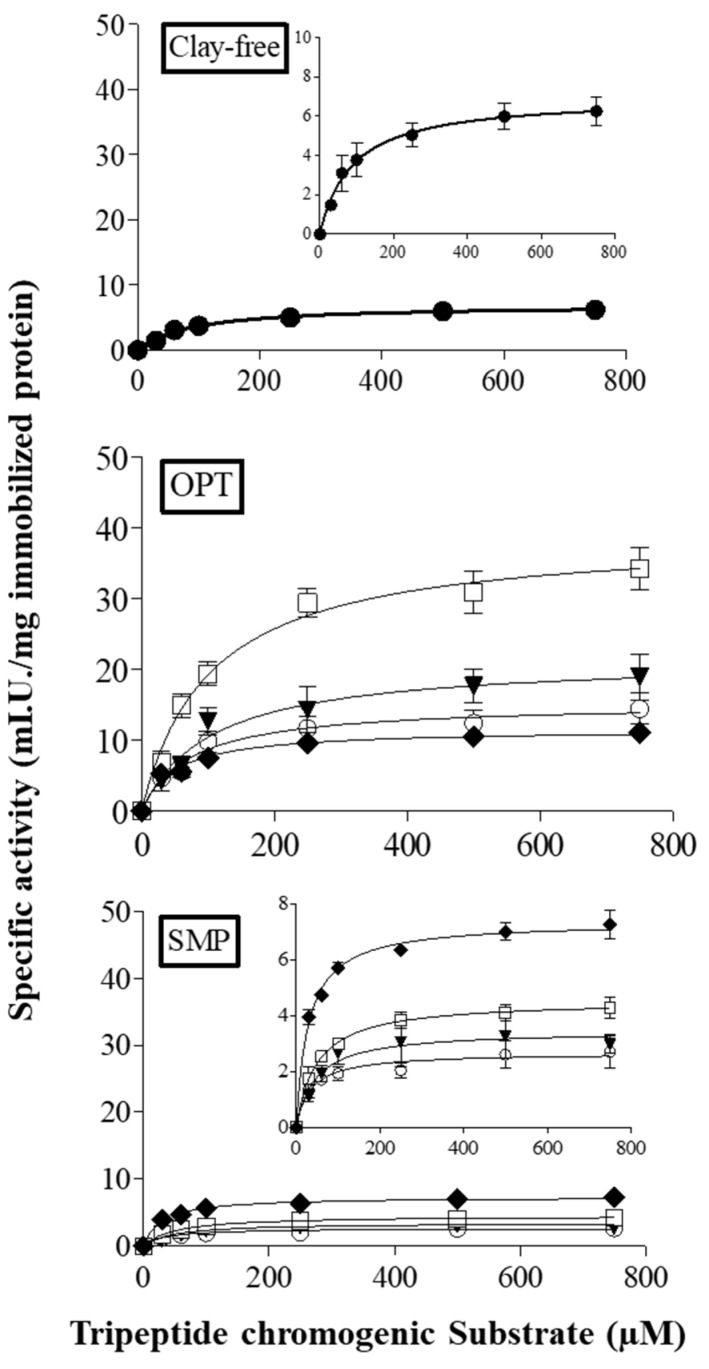
Kinetic curves of papain immobilized on CS–clay composite systems loaded with OPT or SMP at various weight percentages: 20 (○), 30 (▼), 50 (□) and 70 (♦) % *w*/*w*.

The kinetic characterization of the biocatalysts was performed in order to explore the effect of the nanoclays’ inclusion within the polymeric matrix on the behavior of immobilized papain. All the biocatalysts followed the hyperbolic trend described by the Michaelis–Menten equation (Figure 4) and the related catalytic properties are reported in Table 4. Papain on OPT films showed higher V_max_ and k_cat_ values with respect to the enzyme linked to all the other supports (Clay-free and SMP-based films). Notably, the highest V_max_ and k_cat_ values were found for papain immobilized on OPT-50 (38.9 mIU/mg_IP_ and 20,417 min^−1^, respectively), thus indicating a greater product release velocity. However, both parameters significantly increased as the percentage of clay in the nanocomposite increased, except for the ratio 30:70% *w*/*w* (Table 4). Basak et al. [29] described similar results, finding an increased V_max_ value (about +20%) for lipase immobilized on CS–clay (hydrophilic bentonite) with respect to the neat CS support. As widely described in the literature for α-amylase and α-amylase immobilized on clay-CS carriers, an increased V_max_ value may arise from the conformational changes of biocatalysts, which usually occur after the linkage to the support [30].

In contrast, papain immobilized on SMP-20, SMP-30 and SMP-50 films showed significantly lower V_max_ and k_cat_ values with respect to the enzyme linked to Clay-free support, whereas no significant differences were revealed for SMP-70 (Table 4). These results are in agreement with what Cacciotti et al. [14] reported for another protease belonging to the papain-superfamily (bromelain from pineapple stem).

All SMP biocatalysts and OPT-70 showed a significant decrease in K_M_ values (on average −55% and −43% for SMP-based samples and OPT-70, respectively) as compared with Clay-free. No remarkable differences were found for OPT-20, OPT-30 and OPT-50 (Table 4), thus indicating no changes in the enzyme–substrate complex formation in comparison to the reference biocatalyst. Similar results were described by Basak et al. [29], who found comparable K_M_ values for the lipase immobilized on CS and on CS–clay. Moreover, the K_a_ values revealed for papain immobilized on OPT films were 2.4 (OPT-20 and OPT-70) to 5.5-fold (OPT-50) higher as compared with the enzyme immobilized on Clay-free, suggesting a greater apparent affinity of the protease on the aforementioned nanocomposites for the synthetic substrate. Concerning the biocatalysts on the SMP-based supports, their K_a_ values always appeared lower or not statistically different (i.e., SMP-20) with respect to Clay-free film.

Overall, in spite of the lowest IY observed for papain immobilized on OPT-based films, OPT-30 and OPT-50 exhibited the best catalytic performances, both in terms of V_max_, k_cat_ and K_a_, thus indicating a higher efficiency in the release of the reaction product. As known, the decrease in the biocatalyst activity could be ascribed to a great amount of enzyme molecules immobilized on the carrier, since the latter could limit the accessibility of the substrate to the active sites, due to diffusion limitation [31]. Furthermore, as described in the literature [13], the improved catalytic properties observed for papain bound on OPT films could be likely due to the hydrophilic nature of OPT clay, which contributes to create an environment conducive to the interaction between substrate and enzyme. Otherwise, the hydrophobic clay which constitutes SMP-based films, does not facilitate the aforementioned interaction [13].

Thus, among all the produced biocatalysts, papain immobilized on the OPT-50 film was selected as the most suitable to be applied for the protein stabilization treatment of white wines.

### 3.3. Wine Stabilization Treatment in Batch-Scale Stirred Reactor

The partial stabilization of white wines via proteolytic treatment has been already described by other authors [1]. Benucci et al. [20] described the efficacy of a laboratory bench-scale packed-bed reactor, containing papain bound on chitosan beads, in stabilizing white wines.

The effectiveness of papain immobilized on OPT-50 film was tested in a laboratory-scale stirred reactor with the aim of reducing the protein instability of two real white wines (i.e., Manzoni and Sauvignon Blanc), characterized by a different haze potential (601 ± 11 ΔNTU and 116 ± 8 ΔNTU, respectively), as well as by a different initial protein content (465 ± 63 mg_BSAeq_/L and 124 ± 1 mg_BSAeq_/L, respectively). Papain covalently immobilized on OPT-50 nanocomposite film significantly reduced both the haze potential and the protein content in the two wine samples (Table 5). The biocatalyst exhibited the greatest stabilization efficiency in Manzoni wine, with a TRY reduction rate of about 83% and a decrease in total protein amount of approximately 73%. The lowest stabilization efficiency revealed in the Sauvignon Blanc wine (TRY reduction: 31%; total protein reduction: 12%) could be ascribed to the higher content of potential inhibitors found in Sauvignon Blanc with respect to Manzoni wine, especially SO_2_ free (about 2-fold higher in the former compared to the latter wine wine) and the highest alcohol content (about 2% v/v). Indeed, it has been proven that these substances exhibit a strong inhibiting action against the catalytic activity of papain [20].

## 4. Conclusions

The CS-based nanocomposite films produced by the addition of two nanoclays (OPT and SMP) showed improved mechanical properties. The better distribution of OPT clay within the polymeric matrix, as proved by the SEM micrographs and DSC data, imparted a higher Young modulus and σ_max_ to the OPT nanocomposites.

Papain was successfully bound on such carriers, showing the highest efficiency in the release of the reaction product after the covalent immobilization on OPT-30 and OPT-50. The latter biocatalyst reliably reduced both the haze potential and the protein content in the two tested white wine samples, namely Manzoni and Sauvignon Blanc, even if the stabilization efficiency was affected by the content of the potential inhibitors in the wine.

## Figures and Tables

**Table 1:** Oenological parameters of the unfined Manzoni and Sauvignon Blanc wines.

	Manzoni	Sauvignon Blanc
pH	3.37 ± 0.01	3.34 ± 0.01
Total acidity (g/L tartaric acid)	6.00 ± 0.05	6.00 ± 0.05
Alcohol level (% *v*/*v*)	11.2 ± 0.3	13.2 ± 0.2
Free SO_2_ (mg/L)	6 ± 1	10 ± 1
Total SO_2_ (mg/L)	30 ± 2	48 ± 3
Total phenols (mg/L catechin)	217 ± 1	219 ± 4
Total protein (mg/L)	465 ± 43	120 ± 7
ΔNTU Index_0_	600 ± 11	116 ± 8

Δ Nephelometric Turbidity Units (NTU) Index_0_, difference between the turbidity of the initial wine and that of the wine after the heat test.

**Table 2:** Thermal properties of the chitosan and nanoclay-based supports.

	Clay-Free	OPT			SMP		
		20	30	50	20	30	50
T_IHB_ (°C)	121 ± 0.5	118 ± 0.4	124 ± 0.7	111 ± 0.6	118 ± 0.4	115 ± 0.4	105 ± 0.3
Td (°C)	287 ± 1.2	274 ± 1.3	272 ± 0.9	260 ± 1.0	279 ± 1.1	280 ± 1.4	284 ± 1.5
ΔH_mI_ (J/g)	259 ± 1.5	259 ± 1.4	238 ± 1.2	142 ± 0.9	274 ± 1.1	296 ± 1.3	335 ± 2.0

**Table 3:** Mechanical properties of the OPT- and SMP-based films, compared to the neat one (Clay-free).

	E (MPa)	σ_max_ (MPa)	ε_max_
Clay-Free	579 ± 23	16 ± 1	0.30 ± 0.01
OPT-20	738 ± 20	21± 6	0.22 ± 0.08
OPT-30	653 ± 71	23 ± 4	0.13 ± 0.05
OPT-50	1670 ± 202	36 ± 7	0.06 ± 0.03
SMP-20	580 ± 55	24 ± 4	0.28 ± 0.05
SMP-30	528 ± 4	22 ± 3	0.12 ± 0.03
SMP-50	1582 ± 210	19 ± 4	0.04 ± 0.01

**Table 4:** Immobilization yield and kinetic properties of papain immobilized on the CS–clay nanocomposite systems.

Sample	IY (%)	V_max_ (mIU mg^−1^_PI_)	K_M_ (μM)	k_cat_ (min^−1^)	K_a_ (min^−1^ μM^−1^)	R^2^
Clay-free	45 ± 6 ^b,c^	6.9 ± 0.2 ^e^	88 ± 9 ^a^	3183 ± 0 ^e^	36 ± 1 ^d^	0.99
OPT-20	41 ± 4 ^c,d^	15.3 ± 0.9 ^c^	75 ± 16 ^a,b^	6535 ± 1 ^c^	87 ± 4 ^c^	0.97
OPT-30	26 ± 5 ^e^	21.4 ± 1.0 ^b^	106 ± 23 ^a^	12507 ± 1 ^b^	118 ± 6 ^b^	0.98
OPT-50	29 ± 2 ^de^	38.9 ± 2.0 ^a^	102 ± 14 ^a^	20417 ± 2 ^a^	199 ± 3 ^a^	0.99
OPT-70	55 ± 3 ^b^	11.6 ± 0.5 ^d^	50 ± 8 ^b,c^	4225 ± 0 ^d^	85 ± 3 ^c^	0.98
SMP-20	54 ± 7 ^a,b,c^	2.6 ± 0.1 ^f^	34 ± 8 ^c^	1017 ± 0 ^h^	30 ± 2 ^d,e^	0.97
SMP-30	41 ± 5 ^c,d^	3.5 ± 0.2 ^f^	46 ± 11 ^b,c^	642 ± 0 ^i^	14 ± 1 ^f^	0.97
SMP-50	56 ± 2 ^b^	4.5 ± 0.1 ^f^	49 ± 2 ^b,c^	1221 ± 0 ^g^	25 ± 0 ^e^	1.00
SMP-70	60 ± 4 ^a^	7.3 ±0.1 ^e^	29 ± 3 ^c^	2503 ± 0 ^f^	86 ± 1 ^c^	1.00

IY = immobilization yield, V_max_ = maximum velocity, K_M_ = Michaelis–Menten constant, k_cat_ = turnover number, K_a_ = affinity constant. For each parameter, values with different roman letters (a–i) are significantly different according to Tukey’s test (*p* < 0.05).

**Table 5:** Protein stability data of Manzoni and Sauvignon blanc white wines, treated in a laboratory-scale stirred reactor with papain immobilized on OPT-50 carrier at 20 °C (120 rpm, 24 h). Reported data are mean of triplicate measurements.

Treatment	Net Haze after Heat Test		Residual Protein Content	
	ΔNTU	Reduction (TRY, %)	mg _BSAeq_/L	Reduction (%)
**Manzoni**				
Untreated wine	601 ± 11 ^a^	-	465 ± 63 ^a^	-
OPT-50	102 ± 41 ^b^	83	124 ± 35 ^b^	73
**Sauvignon Blanc**				
Untreated wine	116 ± 8 ^a^	-	124 ± 1 ^a^	-
OPT-50	80 ± 1 ^b^	31	109 ± 1 ^b^	12

TRY (%) = Turbidity removal yield expressed as the percentage of haze potential decrease due to the proteolytic treatment. For each parameter, values with different roman letters (a,b) are significantly different according to Tukey’s test (*p* < 0.05).
